# Method of Exhibiting the Iodide of Iron by Extemporaneous Preparation

**Published:** 1854-04

**Authors:** Daniel F. Wright

**Affiliations:** Demonstrator of Anatomy, Memphis Medical College, Tenn.


Method of Exhibiting the Iodide of Iron by Extemporaneous Preparation.—By Daniel F. Wright, M, D., Demonstrator of Anatomy, Memphis Medical College, Tenn.—In view of the difficulty of procuring and keeping a reliable preparation of the Iodide of Iron, I have for some time been in the habit of exhibiting that salt by extemporaneous preparation as follows:

℞. Iodinii,

Ferri Hydrogene Redacti, aa ʒss.,

Mellis, q. s.

Ext. Cinchonæ, ℈ij

Pulv. Glycyrrhizœ, q. s.

Ft. massa et in Pill, xxxij, div.

Rub the iodine and iron together till they form a fine powder; add honey enough to give the consistency of thick molasses; then rub for some time till the fumes of iodine cease to come, and the mixture assumes a greenish tinge. Rub in the extract, and add powder enough to make the mass.

Or make a mixture as follows:

℞. Iodinii, gr. xij.

Ferri Hydrog. Redacti, gr. viij.

Mellis, ʒss.

Ext. Taraxaci, ʒij.

Aq. Menthæ, ℥vj.

M.

Observe the same rule in combining the iodine and iron as in the former prescription, before the water is added.

The average dose is one of the pills or a tablespoonful of the mixture three or four times a day.

It is to be observed that the iron is given in a proportion a good deal larger than would be enough to form the iodide, as the chemical equivalent of iron, being not much more than one fourth that of iodine, gr. viij., of the former would be enough to neutralize ʒss., of the latter. I nevertheless use the quantities as given above, for two reasons; first, because the tonic effects are much increased by the addition of iron in an uncombined state; and secondly, as an effectual method of preserving the salt from decomposition by the absorption of oxygen; this process would be constantly setting iodine free, but by observing such a precaution, the free iodine is constantly taken up by the uncombined iron. For the same purpose, apothecaries have long been in the habit of keeping a mass of metallic iron (as a watch-spring) in their syrups of the iodide of iron.

The directions given for insuring the perfect combination of the elements are highly important, as otherwise the presence of free iodine would be liable to produce a corrosive action on the coats of the stomach.

One or the other of the above formulæ may be used in all cases where the tonic, alterative and diuretic effects of the iodide of iron are indicated. The dose prescribed contains very nearly a grain of the salt, and may, of course, be increased or diminished at the discretion of the physician. I have, myself, used them with considerable success in cases of secondary syphilis, where the vital and assimilative functions have been much impaired by long continued disease. I have found it especially serviceable in those cases of ascites and anasarca, which result from long continued intermittent and remittent fever; in these cases I use the second formula, substituting syr. scillæ ℥i. for the honey. The acetic in that syrup is incompatible with the iodide of iron, but, being present in only small quantities, decomposes only an inconsiderable portion of the salt, which, indeed, the uncombined iron is more than sufficient to restore.—*Am. Jour. of Pharmacy.*

